# Increased Ca^2+^ Sequestration by the Sarco-/Endoplasmic Reticulum in Cardiac Purkinje Cells After Myocardial Infarction

**DOI:** 10.3390/cells15131196

**Published:** 2026-06-30

**Authors:** Ruhul Amin, Zhanné Hopkinson, Louisa Wiede, Kazi T. Haq, Penelope A. Boyden, Henk E. D. J. ter Keurs, Bruno D. Stuyvers

**Affiliations:** 1 Division of BioMedical Sciences, Faculty of Medicine, Memorial University, St. John’s, NL A1B 3V6, Canada; rbio5226@gmail.com (R.A.); zphopkinson@mun.ca (Z.H.); llw013@mun.ca (L.W.); 2 Children’s National Hospital, Washington, DC 20010, USA; khaq@childrensnational.org; 3 Department of Pharmacology, Centre for Molecular Therapeutics, Columbia University, New York, NY 10032, USA; pab4@cumc.columbia.edu; 4 Libin Cardiovascular Institute of Alberta, University of Calgary, Calgary, AB T2N 1N4, Canada; terkeurs@ucalgary.ca

**Keywords:** Purkinje cell, SR-Ca^2+^ uptake, Ca^2+^ arrhythmogenicity, Purkinje-mediated arrhythmia, SERCA, myocardial infarction

## Abstract

During acute coronary occlusion, ischemia is a major determinant of the cell response to subsequent reperfusion and is the major precursor of the typical “ischemia–reperfusion injury” (IRI). Therefore, elucidating the full IRI process primarily relies on a good understanding of ischemia-induced alterations. Ischemic arrhythmias frequently arise during the acute phase of a myocardial infarction (MI) and originate in the terminal arborisations of the cardiac conduction system. These ventricular arrhythmias are triggered by abnormal Ca^2+^-dependent depolarisations (DADs) of Purkinje cells (Pcells) due to increased spontaneous Ca^2+^ release by the sarcoplasmic reticulum (SR). This early alteration of the conduction tissue is also likely to provide a substrate for IRI-related arrhythmogenicity. Recent evidence associates the ischemic phase of the MI with a significant increase in SERCA2 pump expression in Pcells, suggesting that enhanced SR-Ca^2+^ release results from an augmentation of Ca^2+^ sequestration by the SR in those cells. We examined this hypothesis by assessing the impact of ischemia on the dynamics of SR-Ca^2+^ uptake in live Pcells by high-resolution confocal microscopy in a classical canine model of LAD coronary ligation. Pcells from five normal hearts were compared with cells from five hearts 48 Hrs after coronary occlusion. Purkinje-specific Ca^2+^ events, namely peripheral Ca^2+^ wavelets (Wlets) and central cell-wide waves (CWWs), were analysed to assess the regional SR-Ca^2+^ transport of Pcells. A total of 83 normal and 126 MI Wlets, along with 10 normal and 30 MI CWWs, were analysed to compare the peripheral and central SR-Ca^2+^ transports of Pcells between normal and ischemic hearts. Forty-eight hours following the onset of ischemia, individual SR-Ca^2+^ release sites exhibited a 60% increase in Ca^2+^ spark firing rate. However, the site density remained unchanged, indicating an acceleration of intra-SR-Ca^2+^ cycling rather than direct alteration of the SR-Ca^2+^ release channels. While central CWWs remained unchanged, a 37% acceleration of resting Ca^2+^ restoration was readily visible in peripheral Wlets, consistent with enhanced SR-Ca^2+^ uptake at the cell periphery. Computational modelling reproduced these findings when the Ca^2+^ uptake rate was numerically increased by 35%, confirming that augmented SERCA activity is sufficient to explain the pro-arrhythmic SR-Ca^2+^ release of Pcells after MI. Our findings confirm that the augmentation of Ca^2+^ pump density in the periphery of Pcells is associated with an increase in SR-Ca^2+^ uptake, explaining the arrhythmogenicity of Purkinje fibres in an ischemic heart. This ischemia-mediated pro-arrhythmic remodelling of intracellular Ca^2+^ handling in the conduction system is also likely to contribute to triggered activity during subsequent reperfusion.

## 1. Introduction

During coronary occlusion, myocardial ischemia is frequently associated with ventricular tachycardias (VTs), electrical storms, fibrillations (VFs), and sudden cardiac arrests. Although reperfusion limits the infarct size and greatly contributes to cardiac muscle survival, the risk of triggered arrhythmias persists and is a life-threatening component of ischemia–reperfusion injury (IRI). An effective anti-arrhythmic strategy during reperfusion therapies requires a good understanding of the origins of early arrhythmogenesis during the ischemic attack.

Mapping and ablation techniques [1,2] showed that ischemic ventricular tachyarrhythmias (VTs and VFs) arise from ectopic activity in the conduction (His–Purkinje) system [3,4]. Abnormal Ca^2+^ handling of cardiac Purkinje cells (Pcells) has been implicated in this pro-arrhythmic signalling [5,6,7,8,9]. In heart cells, including Pcells, large spontaneous Ca^2+^ waves can depolarise the cell membrane by activating Ca^2+^-dependent currents such as *I_Na-Ca_* and occasionally trigger full action potentials [5,6,10,11,12,13]. Spontaneous Ca^2+^ waves are initiated by the local release of Ca^2+^ by the sarcoplasmic reticulum (SR) and propagate in the cell and cell-to-cell through Ca^2+^ diffusion and a “Ca^2+^-induced Ca^2+^ release” (CICR) process [14]. The spontaneous SR-Ca^2+^ release in cardiac cells is often referred to as “SR-Ca^2+^ leak”. In Pcells, the probability for large electrogenic Ca^2+^ waves increases with the incidence and amplitude of smaller spontaneous Ca^2+^ transients, namely Ca^2+^ sparks and Ca^2+^ wavelets [15]. A consistent intensification of small and large spontaneous Ca^2+^ transients has been reported in Pcells of the dog heart within two days after ligature of the LAD coronary artery [16,17,18]. This confirms the direct relation between increased SR-Ca^2+^ release in cells of the conduction system and lethal tachyarrhythmias in ischemic hearts [6,8,19].

Despite addressing the origin and unpredictability of ischemic arrhythmias and likely providing a determinant of IRI arrhythmogenicity, the fundamental cause of increased SR-Ca^2+^ release in Pcells remains unknown.

In inherited rhythm disorders such as arrhythmogenic right ventricular cardiomyopathy (ARVC) and catecholaminergic polymorphic ventricular tachycardia (CPVT), the pro-arrhythmic increase in SR-Ca^2+^ release is mainly attributed to aberrant ryanodine receptor (RyR) in ventricular myocytes [20,21,22]. Deficient RyR has become the standard interpretation of an abnormal “SR-Ca^2+^ leak” in heart cells and was logically proposed to be the source of abnormal SR-Ca^2+^ release in Purkinje fibres after MI [18]. Nevertheless, a significant increase in SR-Ca^2+^ ATPase expression was recently reported in Pcells of dog, sheep, pig, and human hearts after MI [23], introducing the idea that SR-Ca^2+^ uptake is enhanced in those cells. As supported by computational modelling [15,24] and proposed in [25], greater Ca^2+^ uptake due to the elevation of Ca^2+^ ATPase (SERCA2) activity can increase the rate of Ca^2+^ recycling by the SR and potentiate the SR-Ca^2+^ leak. Here, we verified the hypothesis that increased SERCA expression recently reported at the periphery of Pcells of ischemic hearts [23] is an ischemia-induced molecular alteration directly associated with an increase in SR-Ca^2+^ uptake. To do so, we analysed the impact of ischemia on the regional Ca^2+^ dynamics of live Pcells.

Strong regionalisation has previously been evidenced in the Ca^2+^ mobilisation of Pcells owing to -i- the absence of transverse tubules and -ii- differential expression of SR-Ca^2+^ release channels in the central and peripheral subcellular regions [15,26]. This specificity mediates complex “centripetal” Ca^2+^ mobilisation upon electric stimulation, involving consecutive SR-Ca^2+^ releases from the periphery to the centre of Pcells [24]. In unstimulated conditions, the same compartmentalisation of Ca^2+^ release channels generates region-specific spontaneous Ca^2+^ events, namely wavelets (Wlets), small and rapid waves propagating over short distances at the periphery, and large cell-wide waves (CWWs), travelling longitudinally by diffusion and CICR in the cell centre. We capitalised on the time course of these typical spontaneous Ca^2+^ transients to document the SR-Ca^2+^ uptake function in the different Ca^2+^ release regions of living Pcells in canine hearts with 48-h MI. To focus on the specific cellular response to ischemia, we elected to use a model of permanent coronary occlusion ligation with no reperfusion. A Purkinje-specific model of intracellular Ca^2+^ mobilisation [15,24] was used to confirm that an increase in SR-Ca^2+^ uptake function reproduces the alterations in peripheral Ca^2+^ dynamics detected experimentally in live Pcells after MI.

## 2. Methods

### 2.1. Model of Myocardial Infarction and Cell/Tissue Preparation

Acute myocardial ischemia was induced in healthy adult mongrel dogs (12–15 kg, 1–2 years old and of either sex from Marshall Farm, Concord, MA, USA) by permanent ligation of the left anterior descending (LAD) coronary artery [27,28]. The hearts were explanted 48 h after the ligation. Free-running Purkinje strands were dissected from the subendocardial region of the left ventricle (LV). The method of Pcell isolation was modified from [28,29] and previously described in [23]. Briefly, upon incubation of Purkinje strands with collagenase (Type II, Worthington) at 37 °C, pH 6.7 for 30–40 min, the cells were mechanically dispersed with the same medium with no collagenase, then filtered and finally re-suspended in HEPES-buffered medium with 2 mM Ca^2+^ and pH 7.3. The cells were used the same day for intracellular Ca^2+^ imaging. Normal (control) and “MI” Pcells were prepared from PFs of non-infarcted hearts and hearts with 48-h MI. MI Pcells were isolated from PFs located in the subendocardial region of the left ventricular chamber, including the infarction zone.

The institutional animal care committees of Memorial University of Newfoundland, the University of Calgary, and Columbia University have approved the utilisation of animals as described in the protocols. This practice complies with the policies of experimental animal practice in Canada and USA.

### 2.2. Intracellular Ca^2+^ Imaging and Analysis of Spontaneous Ca^2+^ Transients

**Figure 1:**
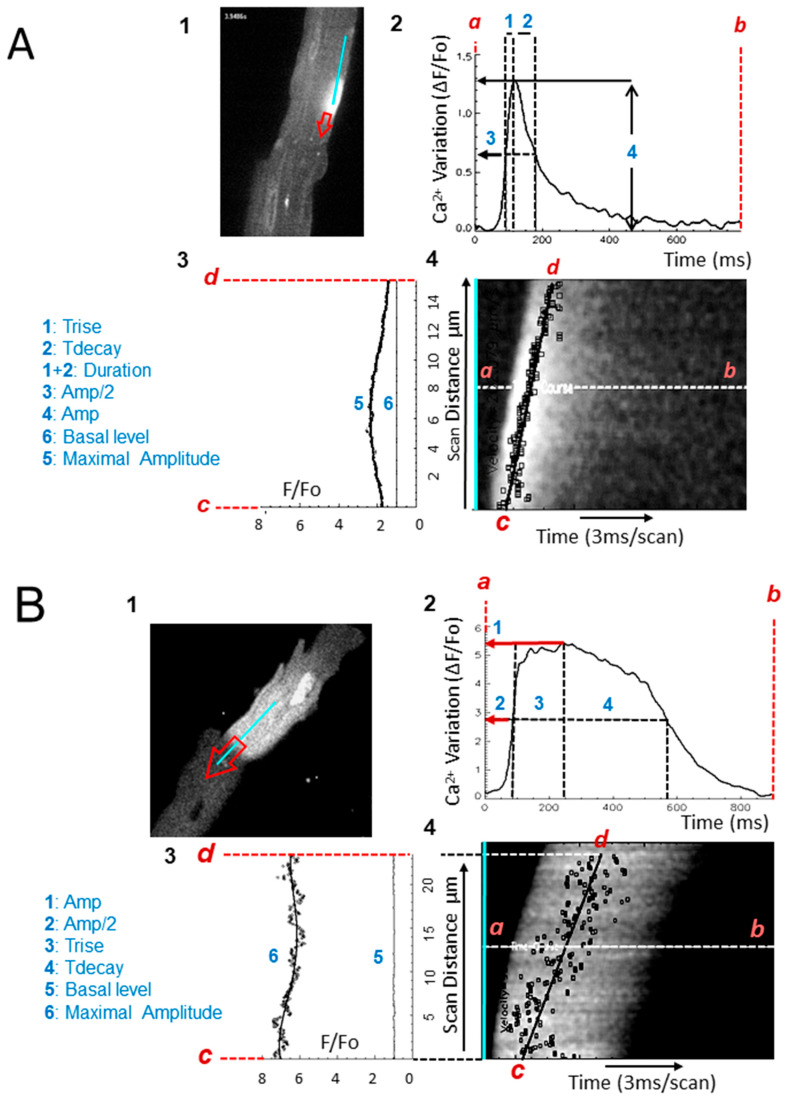
High-resolution confocal microscopy and analysis of spontaneous Ca^2+^ transients in dog Purkinje cells. (**A**) Wavelet transients were captured by fluorescence laser scanning confocal microscopy (LSCM) at a scanning rate of 350 Hz along 25–50 µm distances. As reported previously, wavelets propagated exclusively at the periphery of Pcells in the “SubSL compartment” [15,30]. For clarity, a scan example (blue line) of the peripheral region is represented on a representative 2D (spinning disk) confocal snapshot of a propagating wavelet in (**A.1**). A total of 250 successive scans of the same region (3 ms/scan) were arranged into the scan-line image shown in (**A.4**). The time course of the transient shown in (**A.2**) was measured at half the distance of the wave’s overall propagation along the a-b segment represented in (**A.4**). A linear regression through maximal scan values verified the linearity of the propagation (see segment c-d in (**A.4**)) and consistency of the transient amplitude (**A.3**) during the propagation. The data points represented in (**A.4**) are the maximal values measured in the scans. Transients with amplitude fluctuation > 5% during the propagation were rejected. The parameters listed in (**A.3**) were measured as indicated in (**A.2**) and (**A.3**): Trise is the time of the rising phase at half-maximal amplitude of the time course measured along segment a-b and expressed in ms; **Tdecay** is the decay duration at the half-maximal amplitude of the time course measured along the segment a-b and expressed in ms; **Amp** is the maximal amplitude of the time course and expressed in Fluo-4 fluorescence F/Fo ratio units (see methods); **basal level** is the resting level of Ca^2+^ measured during the overall propagation during the scan and measured in F/Fo ratio units; **maximal amplitude** is the maximal amplitude detected during the overall propagation during the scan and expressed in F/Fo ratio units. A custom module of IDL software automatically generated data for (**A.2**–**4**) from raw scan line images shown in (**A.4**). (**B**) CWW transient parameters were measured using the method described for wavelet transients in (**A**). Scans were positioned in the centre of the cells to capture the exact central kinetics of the Ca^2+^ variation. For clarity, an example of a central scan is superimposed on a 2D confocal snapshot of a representative CWW propagation along the cell in (**B.1**).

After isolation, Pcells were placed in a field-stimulated experimental chamber and perfused with 2 mM Ca^2+^ standard HEPES solution (Tyrode) at 37 °C (pH 7.3) for 5 min. The cells were successively incubated with 5 µM Fluo4-AM (ThermoFisher Scientific, Waltham, MA, USA), rinsed, and left in the dark for 10 min. The cells were paced at low frequency (0.1 Hz) to uniformly activate intracellular Ca^2+^ cycling throughout the cells in the preparation. A Fluo-4-loaded Pcell was selected in each preparation (1 cell/preparation) based on a positive response to stimulation (cell shortening) and criteria initially defined in [28]. Ca^2+^-dependent fluorescence of Fluo-4 was captured by line scanning confocal microscopy (LSCM) at 333 scans/sec for quantitative analysis or 2-dimensional spinning disk confocal microscopy (2DCM) at 30 frames per second (fps) for qualitative assessments as described elsewhere [15,18,30,31]. The relative free-Ca^2+^ concentration ([Ca^2+^]) was given by the pixel-to-pixel ratio F/Fo, where F and Fo are the instantaneous fluorescence intensity and basal fluorescence intensity, respectively. Image processing and Ca^2+^ transient analyses used custom IDL programs (L3Harris Geospatial Solutions, Inc., Broomfield, CO, USA) and modules of the open-source NIH software Image J (1.54g). Individual spontaneous Ca^2+^ transients were analysed as described in Figure 1.

### 2.3. Modelling of Ca^2+^ Transients

To assist the interpretation of data from live cells, we used a computational model previously developed to predict intracellular Ca^2+^ mobilisation in cardiac Purkinje fibres [15,24,32].

The modelling principles are summarised in the Supplementary Materials.

### 2.4. Statistics

We considered that Pcells isolated from 5 normal hearts were representative of a non-ischemic (normal) cellular environment, and cells from 5 hearts with MI were representative of the cell population in typical hypoxic/ischemic conditions. In Table 1, the time course parameters of Ca^2+^ transients were measured from individual waves and averaged for normal and MI cell groups. The data are expressed as the mean ± SD and were compared by unpaired *t*-test and multi-parametric variance analysis.

## 3. Results

### 3.1. Alteration of Individual Ca^2+^ Release Sites in Pcells After MI

**Figure 2:**
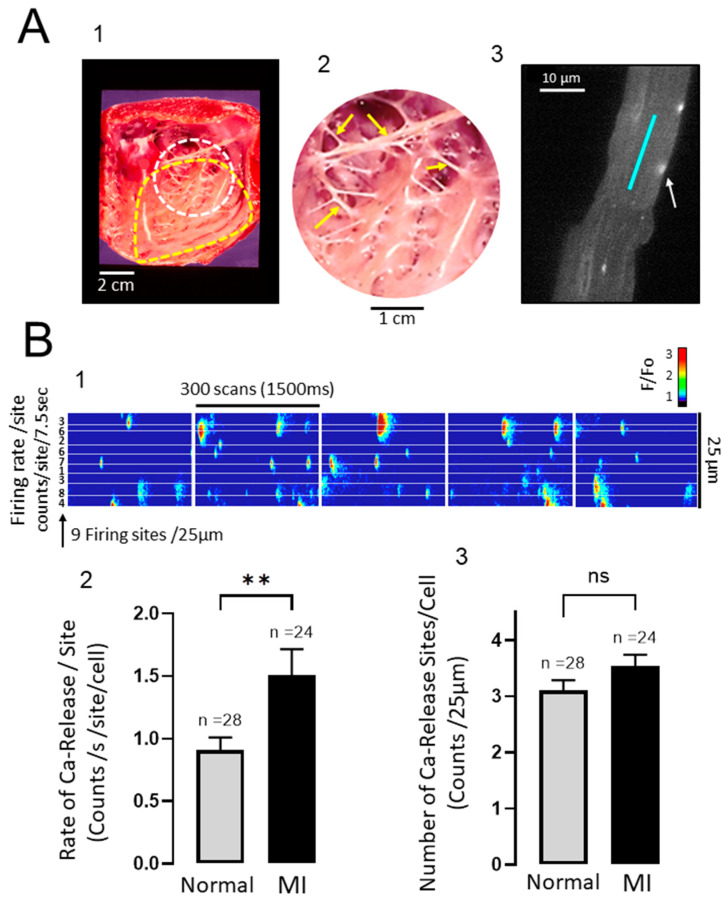
**Ca^2+^ spark activity of individual Ca^2+^ release sites in Pcells of canine hearts with acute ischemic MI.** (**A**) Pcells were isolated from the left ventricle of normal dog hearts and dog hearts with 48-h MI. Permanent LAD coronary occlusion induced typical transmural MI within less than two days, with no evident sign yet of scar tissue (see yellow dashed line in (**A.1**)). At this stage, we postulated that the MI zone comprised myocardial cells undergoing typical acute ischemia. Pcells were isolated from endocardial free-running Purkinje strands (see dashed white circle in (**A.1**) and yellow arrows in (**A.2**)). (**A.3**) illustrates the capture of spark activity by a 25 µm line scan (5 ms/scan) positioned randomly in the cell; for clarity, a scan line is represented on the 2D confocal (spinning disk) image of a representative Fluo-4-loaded Pcell. (**B**) Consecutive scans were plotted as shown in (**B.1**); the spark firing rate (**B.2**) and density (**B.3**) of active Ca^2+^ release sites were measured as indicated in (**B.1**). To limit photobleaching, scanning was interrupted and cell illumination was occulted for 5 ms every 1.5 s (300 scans). The firing rate (**B.2**) and number of sites (**B.3**) were typically captured by 7.5 s scanning sequences as indicated in (**B.1**) and were compared between Pcells of normal hearts (Norm: n = 28 cells from 5 normal hearts) and Pcells of hearts with MI (MI: n = 24 cells from 4 MI hearts). Data are expressed as the mean ± SD; **: significant difference (*p* < 0.01); ns: no difference.

Purkinje fibers (Figure 2A.2) were dissected from normal ventricles and from ventricles with 48 h of ischemic MI (Figure 2A.2). We compared the activity of individual Ca^2+^ release sites between normal and MI Pcells by measuring the spark firing rate of each site identified as active along 25–50 µm line scans positioned randomly within the cells (Figure 2A.3,B.1). Consistently with the upper shift of the overall spark rate versus pCa relationship reported earlier in canine Pcells after MI [18], we found that, on average, the individual Ca^2+^ release sites fired sparks at 60% greater frequency in MI Pcells (1.5 ± 1.0 spark counts/site/s, n = 24 cells from N = 4 hearts with 1 scan sequence per cell) compared with normal Pcells (0.9 ± 0.5 spark counts/site/s, n = 28 cells from N = 5 hearts with 1 scan sequence per cell, *p* < 0.01) (Figure 2B.2). No difference in the number of active sites per scan was detected between the two groups [3.12 ± 0.96 (Norm; n = 28 cells) versus 3.54 ± 0.98 counts/scan/cell (MI; n = 24 cells; *p* = 0.112)] (Figure 2B.3).

### 3.2. Region-Specific Alterations of Propagating Ca^2+^ Transients

**Figure 3:**
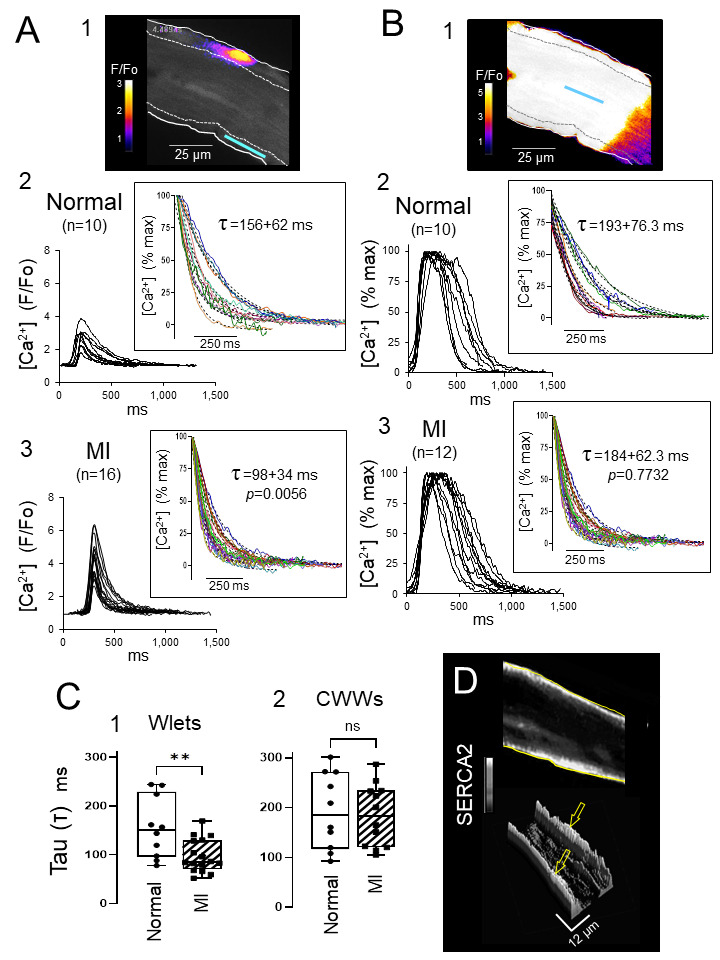
**Comparisons of normal vs. MI Pcells of peripheral (Wlets) and central (CWWs) Ca^2+^ removal.** (**A**) Typical Wlet transients were captured by 25 µm line scans positioned in the peripheral regions of Pcells as indicated (blue line) in the representative 2D confocal snapshot in panel (**A.1**); the peripheral regions of the cell are underlined to emphasise the regionalisation of Wlet occurrence. The time courses of 10 normal Wlets (from 6 cells) and 16 MI Wlets (from 6 cells) are displayed in panels (**A.2**) and (**A.3**), respectively. The mean characteristics of the waves are reported in Table 1. After normalisation to the maximal Ca^2+^ concentration, the decay phase of each transient was isolated and represented in the insets of (**A.2**) and (**A.3**). The following one-phase exponential decay model was used to fit the data of each transient: [Ca^2+^] = (Y0 − P)*exp[-(1/τ) * t] + P, where Y0 is the [Ca^2+^] value at t = 0, P is the [Ca^2+^] value at infinite t, and τ is the time constant of the decay expressed in ms and reflects the rate of intracellular Ca^2+^ decline. The averaged τ values (mean ± SD) are reported on the graphs for each group. (**B**) Individual CWWs were sampled by LSCM as indicated in the representative typical confocal snapshot of (**B.1**) and analysed as reported in Table 1. The time courses of 10 normal and 12 MI CWWs are represented in (**B.2**) and (**B.3**), respectively; the Ca^2+^ decay phases of the waves are displayed in the insets. The same exponential model was used to estimate and compare the rates of Ca^2+^ decline (τ) between the normal and MI CWWs. (**C**) The time constants τ of the Ca^2+^ decay of Wlets (**C.1**) and CWWs (**C.2**) were compared between the normal and MI Pcells represented in panels (**A.2**,**B.2**) and (**A.3**,**B.3**), respectively; **: significant difference (*p* < 0.01; unpaired *t*-test), ns: no difference. (**D**) Some cells were stained with the SERCA2 antibody used in [23]. As shown in 2D and 3D in a typical MI cell, the fluorescent staining revealed the same peripheral elevation of the Ca^2+^ pump density under the membrane (see yellow arrows); the cell boundary is underlined in yellow.

As previously reported [15,30], two different types of spontaneous Ca^2+^ waves were observed in Pcells: local small-amplitude Ca^2+^ waves (Wlets), propagating on short distances at the cell periphery (Figure 3A), and large-amplitude cell-wide waves (CWWs), spanning the full width and propagating in the longitudinal direction of the Pcells (Figure 3B).

As reported in Table 1, the Wlets exhibited 53% larger amplitude and a 23% decrease in their total duration due to a 25% shortening of the Ca^2+^ decay phase after MI. Figure 3A.2 and A.3 show that fitting a one-phase exponential function through the decline in Ca^2+^ revealed a significant 37% reduction in the time constant (τ) of the Ca^2+^ decay of Wlets. This change in τ was not related to the larger transient amplitude (amp) since the same observation was made after normalisation to the maximal amplitude (see panel c inset in Figure 3A2,A3). Also, after MI, we found that Wlets propagated at 22% lower velocity compared to normal Wlets (Table 1), ruling out the propagation from the causes of shorter transient.

At 2 mM Ca^2+^ and 35 °C, CWWs were seen at a very low rate (approximately one wave every 4–6 s) in the core of both normal and MI Pcells. The analysis of the wave time course and propagation, as illustrated in Figure 1, revealed no alteration in the duration, amplitude and/or velocity of CWWs after MI (Table 1). Fitting the decline in Ca^2+^ of CWWs through the same model of exponential decay used for Wlet transients showed no difference in the time constant (τ) between normal and MI Pcells after MI (see insets in Figure 3B.2, B.3). Interestingly, staining representative Pcells of ischemic hearts with the same SERCA2 antibody used previously in [23] showed an identical increase in SR-Ca^2+^ pump expression over the peripheral region (Figure 3C), where Wlets exhibited an acceleration of their Ca^2+^ decay (Figure 3A).

### 3.3. Predicted Impact of Increased SR-Ca^2+^ Uptake on SR-Ca^2+^ Release

**Figure 4:**
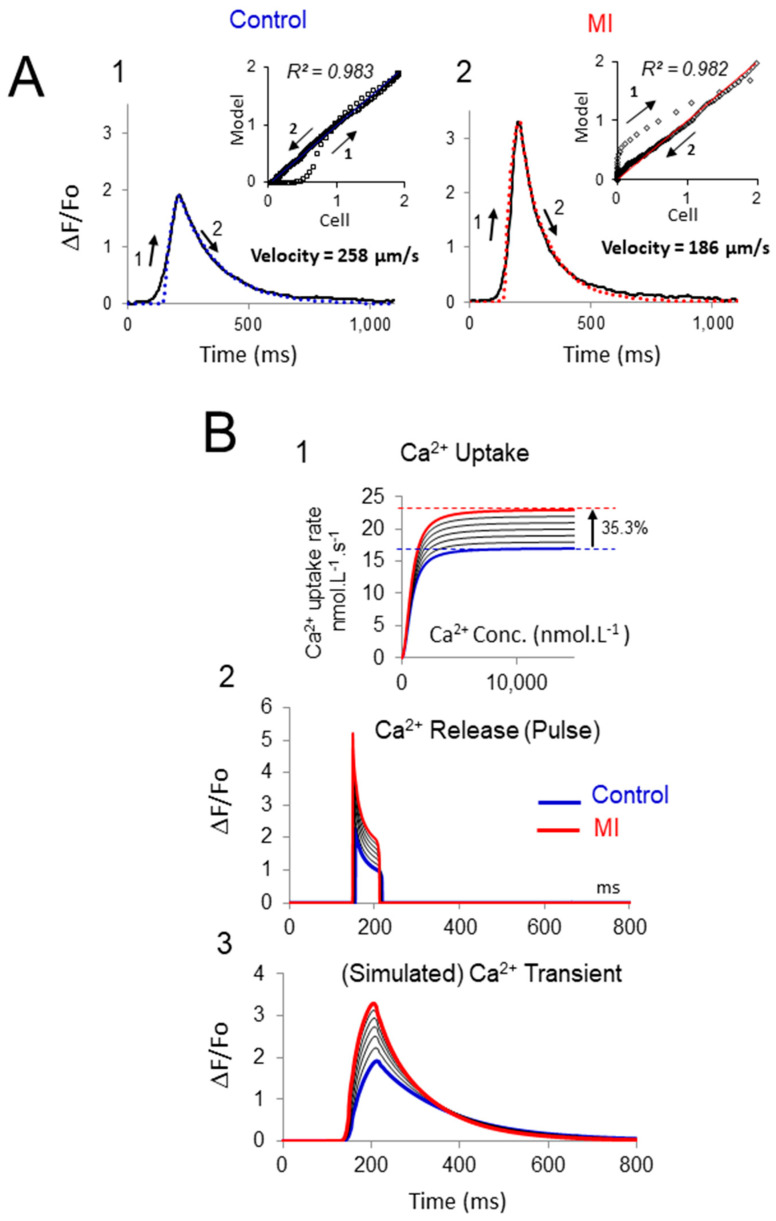
**Computational evidence of SR-Ca^2+^ uptake’s contribution to the alterations in peripheral Ca^2+^ dynamics in Pcells after MI.** (**A**) Ten and 17 representative Wlets were combined, respectively, to produce the averaged Ca^2+^ transients of normal (control) (**A.1**) and MI (**A.2**) Pcells. The Purkinje-specific model of Ca^2+^ mobilisation described in the Supplementary Materials and references [24,32] was used to replicate the normal and MI-averaged transients; predicted (dotted line) and experimental (plain line) curves are superimposed in (**A.1**) and (**A.2**). The accuracy of the predictions was estimated by linear regressions as indicated in the corresponding insets, where arrows 1 and 2 indicate the rising and decay phases of the transients, respectively. (**B**) The maximal Ca^2+^ uptake rate (*Umax*) was incrementally increased (**B.1**) in the numerical expression of the normal Ca^2+^ transient shown in (**A.1**); the simultaneous effects of Umax increments on the Ca^2+^ release pulse function and overall Ca^2+^ transient are represented in (**B.2**) and (**B.3**), respectively. As indicated in (**B.3**), a complete switch from normal transient (blue trace) to MI transient (red trace) was achieved with a ~35% increase in Umax (see (**B.1**)). Ca^2+^ variations are expressed as variations in the F/Fo ratio from 1 (ΔF/Fo); one variation unit is equivalent to 100 nmol.L^−1^ of free Ca^2+^ [15].

Unlike CWWs, Wlets exhibited a significant decrease in τ, revealing consistent acceleration of the Ca^2+^ decay phase after MI. As discussed further in our report, this acceleration is likely to reflect an increased rate of SR-Ca^2+^ uptake in the cell periphery. Nevertheless, as reported in Table 1, the Wlets exhibited a simultaneous increase in amplitude and decrease in propagation velocity. To examine whether these alterations were compatible with the local increase in SR-Ca^2+^ uptake, we used a computational model initially developed to mimic the typical Ca^2+^ mobilisation of Pcells [15,24] (see Supplementary Materials). As illustrated in Figure 4A, we first verified that the model could faithfully reproduce the time courses of normal and MI Wlets shown in Figure 3. As reported in the graphs of Figure 4A, the calculations also indicated that switching from normal to MI transients would simultaneously reduce the propagation velocity by 27% (from 258 to 186 µm/s). In Figure 4B, the model predicted the impact of increasing SR-Ca^2+^ uptake on the Ca^2+^ release (“Ca^2+^ pulse”) function (B.2) and the transient time course (B.3) of peripheral Ca^2+^ transients. The maximal Ca^2+^ uptake rate (*Umax*) used in the simulation of a normal Ca^2+^ transient in Figure 4A.1 was incrementally increased (B.1) until the Ca^2+^ uptake rate–Ca^2+^ relationship matched that required to reproduce the MI transient of Figure 4A.2. The other parameters of the uptake rate–Ca^2+^ relationship, namely the Hill coefficient (*N_Hill_*) and *EC*_50_, were kept constant during the test. Figure 4B shows that each *Umax* increment (B.1) was accompanied by a simultaneous rise in the amplitude of the Ca^2+^ release pulse (B.2) and transient amplitude (B.3) of Wlets. As indicated in Figure 4B.1, a 35% augmentation of *Umax* could reproduced the MI Wlet time course. No alteration of other model input parameters, such as the diffusion coefficient or Ca^2+^ release threshold, was necessary for successful simulation.

## 4. Discussion

### 4.1. Spontaneous Ca^2+^ Events to Probe the Regional Ca^2+^ Dynamics of Pcells

Our current study focused on spontaneous rather than electrically evoked Ca^2+^ transients. In Pcells of large mammalian species, different Ca^2+^ release channels are expressed in distinct concentric layers of the SR [15]. In response to electric stimulation, this regionalisation of SR-Ca^2+^ release generates complex centripetal Ca^2+^ mobilisation [24], whereby the evoked Ca^2+^ transient arises under the sarcolemma and propagates to the centre by a *CICR–diffusion–CICR* process. During centripetal propagation, the Ca2+ transient overlaps with successive layers of Ca2+ release, making it difficult to delineate and characterise regional Ca^2+^ dynamics under stimulated conditions. In contrast, under unstimulated conditions, the Ca^2+^ release channels open spontaneously and generate Ca^2+^ transients specific to each region [15,30], namely, Wlets at the cell periphery and CWWs in the core. Computational studies demonstrated that combinations of SR-Ca^2+^ release, Ca^2+^ diffusion, and Ca^2+^ uptake can reproduce the Ca^2+^ transients of Pcells [15,24], which, conversely, can be dissected to document the individual cellular functions. Here, we analysed the Wlets and CWWs to “probe” the Ca^2+^ uptake function and examine whether Pcells exhibit a change in the peripheral region after MI as was recently hypothesised from increased regional SERCA density [23].

### 4.2. Acceleration of SR-Ca^2+^ Recycling: A Cause of Larger Ca^2+^ Leak in Pcells After MI

An upward shift in the relationship between the incidence of Ca^2+^ sparks and Ca^2+^ concentration (pCa) has been reported in dog Pcells after MI [18]. This finding clearly indicated an augmentation of spontaneous SR-Ca^2+^ release in those cells, which could explain the (Ca^2+^-mediated) arrhythmogenicity of Purkinje fibres in ischemic hearts [8]. This larger spontaneous Ca^2+^ release was first interpreted from an elevation in the sensitivity of SR-Ca^2+^ release channels to Ca^2+^ [18], possibly due to a decrease in the luminal Ca^2+^ release threshold [20]. Nevertheless, an increase in the Ca^2+^ sensitivity is expected to activate more abundant SR-Ca^2+^ channels for the same SR-Ca^2+^ level. Here, we found no change in the recruitment of active Ca^2+^ release sites. In addition, contrasting with the ubiquitous RyR2 channel of ventricular myocytes, three different forms of SR-Ca^2+^ release channel (RyR2, RyR3, InP_3_R1) are expressed in distinct regions of Pcells [15,30]. Though not completely excluded, simultaneous alteration of the three channels within 48 h is less likely. Alternatively, acceleration of Ca^2+^ recycling by the SR is expected to increase the opening frequency of a constant number of SR-Ca^2+^ channels, without altering channel Ca^2+^ sensitivity. Our spark analysis supports this explanation, which is also consistent with [25].

### 4.3. Increased SR-Ca^2+^ Uptake and Faster SR-Ca^2+^ Recycling After MI

Two conditions can increase the circulation rate of free Ca^2+^ in the SR compartment and accelerate Ca^2+^ cycling by the SR: a reduction in Ca^2+^ buffering by the reticular proteins, such as CASQ2 [33], and augmentation of SR-Ca^2+^ uptake [34]. It was previously shown in canine Pcells that the caffeine-induced Ca^2+^ transient was unchanged after MI [18]. This indicates that, in steady state, the releasable amount of Ca^2+^ in the SR is not affected after MI, thereby excluding Ca^2+^ buffering as a cause of faster SR-Ca^2+^ cycling and, conversely, supporting the implication of SR-Ca^2+^ uptake.

### 4.4. Peripheral Location of SR-Ca^2+^ Uptake Elevation

A recent study revealed that the density of SR-Ca^2+^ ATPases (SERCA2) is consistently increased at the edge of Pcells; this was verified in dog, sheep, pig and human ischemic hearts, supporting the translational interest of our findings [23]. The faster Ca^2+^ decay of Wlets observed in our current study indicates a peripheral augmentation of SR-Ca^2+^ uptake, matching the greater expression of SR-Ca^2+^ pumps in this region. In contrast, the absence of modification in the decay of CWWs seems to exclude alteration of SR-Ca^2+^ uptake in the core of Pcells, where, supportively, no change in the density of Ca^2+^ pumps was detected [23]. We conclude that ischemic conditions in the heart selectively affect Ca^2+^ handling at the periphery of Pcells, i.e., in the region where small Ca^2+^ events have been shown to trigger large depolarising CWWs [15].

### 4.5. An Increase in Peripheral SR-Ca^2+^ Uptake in Pcells: A Primary Consequence of Ischemic Conditions

Our current conclusion that peripheral SR-Ca^2+^ uptake is increased after MI is based on the faster Ca^2+^ decline of the Wlet transient. However, this alteration was accompanied by a 50% increase in the amplitude and a 22% decrease in the propagation velocity of Wlets, raising the question of whether these Ca^2+^ changes are secondary to the increase in SR-Ca^2+^ uptake or primary consequences of other anomalies in Pcells. Our numerical model predicted that a 35% increase in the rate of Ca^2+^ uptake could reproduce the change of normal into MI Wlet transients, and this switch would be associated with a reduction in the Wlet velocity comparable to that seen in live Pcells after MI (Table 1). As described in [25], mechanistically, stronger Ca^2+^ pumping is anticipated to slow down the process of *CICR–diffusion–CICR* responsible for the progression of the wave front and, therefore, could explain the slower propagation of Wlets after MI. The large increase in the Wlet transient amplitude was also predicted when the rate of Ca^2+^ uptake was enhanced in the model. This predicted larger amplitude was in agreement with the augmentation of the Ca^2+^ pulse shown in Figure 4B.2, indicating that the rise in the amount of Ca^2+^ released from the SR is a direct consequence of increased Ca^2+^ re-pumping in the SR.

### 4.6. The Nature and Impact of Larger SR-Ca^2+^ Uptake at the Cell Periphery

Our analysis of Wlets revealed a selective increase in SR-Ca^2+^ uptake at the periphery of Pcells in the ischemic heart. An augmentation of SERCA2 expression in this region, as reported recently under identical conditions [23], can explain this functional Ca^2+^ alteration at the periphery.

It is logical to anticipate that additional expression of Ca^2+^ pumps will potentiate the overall SR-Ca^2+^ uptake function and increase the rate of electrogenic spontaneous SR-Ca^2+^ releases in Pcells. Nevertheless, besides their density, the nature of the Ca^2+^ pumps may also contribute to the alteration of SR-Ca^2+^ uptake at the Pcell periphery after MI. The SERCA2a sub-isoform represents more than 95% of SR-Ca^2+^ pumps in mammalian heart cells [35,36] and is produced by pre-mRNA splicing of the SERCA2 gene *ATP2a2* [36,37]. The splicing process is tissue-specific, generating the SERCA2a sub-isoform in the heart and SERCA2b in virtually all other non-cardiac tissues [37]. Importantly, predominant SERCA2b expression at the cell periphery has been reported in other tissues such as smooth muscle of the pulmonary artery [38], and the SERCA2b transcript has been found to increase by 180% in Purkinje fibres of pig hearts with 48 h of ischemic MI [23]. We propose that the increased SR-Ca^2+^ uptake evidenced in our current study is directly associated with the expression of the ubiquitous SERCA2b pump at the periphery of Pcells after MI.

### 4.7. Limitations

Our imaging protocol prioritised cell survival and the spatiotemporal resolution of individual transients, notably by using the line scanning technique, which limits the laser illumination duration and intensity on cells and maximises the sampling rate of Ca^2+^ variations. This procedure did not permit us to evaluate the frequency of low-incidence electrogenic CWWs and did not, therefore, confirm the Ca^2+^ arrhythmogenicity of Pcells in our current study. Similarly, the confocality did not permit measuring the Wlet frequency due to the extreme localisation, random occurrence and short life span of these events. Nonetheless, an increase in the rate of depolarising CWWs associated with an augmentation of small peripheral events was previously demonstrated in the same model of Purkinje fibres [6,16,17]. Because our highly focused present study is the logical extension of those observations, a re-validation of the Ca^2+^ arrhythmogenicity of Pcells in our canine model of ischemia was not necessary.

### 4.8. Conclusions

In the dog model of coronary occlusion, myocardial ischemia is accompanied by an acceleration of SR-Ca^2+^ uptake in the periphery of Pcells. This regional Ca^2+^ alteration could be correlated with the expression of the SERCA2b pump in the same region and may provide the molecular basis of Purkinje arrhythmogenicity in the heart with ischemic MI. Ca^2+^ overload, a known component of reperfusion injury, may accentuate the effect of increased SR-Ca^2+^ uptake on pro-arrhythmic SR-Ca^2+^ release and promote further triggered activity, at least in the His–Purkinje system.

## Figures and Tables

**Table 1:** **A comparison of Ca^2+^ waves between normal and post-MI Pcells.** Samples of Wlets and CWWs were captured by LSCM from 15 Pcells isolated from 5 normal dog hearts and 10 Pcells from 5 hearts with 48Hrs coronary ligation. The time course parameters and velocity of the Wlets and CWWs were averaged and compared between the normal and MI groups. Each parameter is expressed as the mean ± SD. The differences between MI and normal conditions were considered significant when *p* < 0.01 (*) and *p* < 0.0001 (**). To facilitate the description and interpretation of the results, the significant differences are also expressed as a percentage of the control (normal) values; amp is the mean amplitude of the transient expressed in Fluo-4 fluorescence variation units ΔF/Fo; Trise is the time of the rising phase at half-maximal amplitude in ms; Tdecay is the time of the exponential decay phase at half-maximal amplitude in ms; Duration is the total duration at half-maximal amplitude in ms.

		Amp ΔF/Fo	Velocity µm/s	Trise ms	Duration ms	Tdecay ms
**Wavelets**	**Normal** (N = 5 hearts; 15 cells; n: nb of waves) (mean ± SD)	1.19 ± 0.98 (n = 83)	237 ± 150 (n = 79)	48 ± 28 (n = 83)	154 ± 72 (n = 83)	106 ± 51 (n = 83)
	**MI** (N = 5 hearts; 10 cells; n: nb of waves) (mean ± SD)	1.80 ± 1.14 ** (n = 126)	186 ± 90 * (n = 124)	38 ± 19 * (n = 126)	118 ± 49 ** (n = 126)	80 ± 36 ** (n = 126)
	*p* (%Normal)	<0.0001 (53%)	0.007 (−22%)	0.003 (−21%)	<0.0001 (−23%)	<0.0001 (−25%)
**CWWs**	**Normal** (N = 5 hearts; 15 cells; n: nb of waves) (mean ± SD)	5.26 ± 2.49 (n = 14)	102.9 ± 50.7 (n = 12)	127.5 ± 82.4 (n = 14)	409.5 ± 167.0 (n = 14)	282.0 ± 144.8 (n = 14)
	**MI** (N = 5 hearts; 10 cells; n: nb of waves) (mean ± SD)	4.197 ± 2.234 ns (n = 30)	135.4 ± 86.28 ns (n = 28)	103.3 ± 53.13 ns (n = 30)	317.1 ± 114.8 ns (n = 30)	213.8 ± 76.17 ns (n = 30)
	*p*	0.931	0.993	0.998	0.406	0.739

## Data Availability

Data will be made available upon reasonable request.

## Supplementary Materials

The following supporting information can be downloaded at: https://www.mdpi.com/article/10.3390/cells15131196/s1.
