# Supplementary material for: Increased Ca2+ Sequestration by the Sarco-/Endoplasmic Reticulum in Cardiac Purkinje Cells After Myocardial Infarction
Source: Cells. 2026 Jun 30;15(13):1196. doi: 10.3390/cells15131196 (PMC13359873; doi:10.3390/cells15131196)
Supplement: Supplementary file 1 [file cells-15-01196-s001.zip › cells-4383221-supplementary.pdf]

Modelling Principles.

A-

$$\frac{d\,[Ca\,]}{dt} = D\frac{1}{x}\,d\left(x\,\frac{d\,[Ca\,]}{dx}\right)\frac{1}{dx} + R - U$$
$$- k_{+}^{ATP}\left(\,[\,ATP\,] - [\,Ca\,.\,ATP\,]\,\right)[\,Ca\,] + k_{-}^{ATP}\,[\,Ca\,.\,ATP\,]$$
$$- k_{+}^{TnC}\left(\,[\,TnC] - [\,Ca.\,TnC]\,\right)[\,Ca] + k_{-}^{TnC}\,[\,Ca.\,TnC]$$
$$- k_{+}^{CaM}\,([CaM\,] - [Ca\,.CaM\,])\,[Ca] + k_{-}^{CaM}\,[Ca\,.CaM\,]$$
$$- k_{+}^{Fluo4}\,([Fluo4\,] - [Ca\,.Fluo4])\,[Ca\,] + k_{-}^{Fluo\,4}\,[Ca\,.Fluo\,4]$$

B-

Ca^{2+} Diffusion

D (in water) = 3.10^{-6} cm^2.s^{-1}

C-

Ca^{2+} Binding/Dissociation

$$Ca^{2+} + Ligand \underset{k_{-}^{Ligand}}{\overset{k_{+}^{Ligand}}{\rightleftarrows}} Ca^{2+}\text{-}Ligand$$

Ligands (L): Fluo4, ATP, TnC, CaM

D-

Ca^{2+} Release Function (*R*): *"Pulse Shape"*

If [Ca^{2+}] in the node > Ca $^{2+}$Threshold Thr

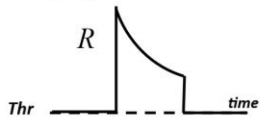

$$R = a(1 - e^{-(t-t_s/\tau_{on})}).e^{-(t-t_s/\tau_{off})}.\frac{[Ca]_{\mathrm{Rest}}}{[Ca]_t}$$

E-

Ca^{2+} Uptake Function (*U*): *"Sigmoidal Shape"*

$$U = \frac{U_{MAX}\cdot[Ca]_N^{Hill}}{EC_{U_{50}}^{Hill} + [Ca]_N^{Hill}}$$

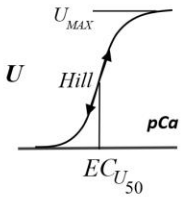

F- Parameters, variables and units

t	*time (s)*
t_s	*start time (s)*
a	*amplitude of the Ca^{2+}-release (mol/L)*
τ_{on}	*on-time constant (s)*
τ_{off}	*off-time constant (s)*
$[Ca]_{Rest}$	*Resting $[Ca^{2+}]$ (nmol/L)*
$[Ca]_t$	*instantaneous $[Ca^{2+}]$ (nmol/L)*
$[Ca]_N$	*$[Ca^{2+}]$ in the node (nmol/L)*
R	*Ca^{2+}- release flux (number of Ca^{2+} ions/s)*
U	*Ca^{2+}- uptake flux (number of Ca^{2+} ions/s)*
U_{MAX}	*maximal Ca^{2+}- uptake flux (number of Ca^{2+} ions/s)*
EC_{U50}	*Ca^{2+} at half maximal uptake (nmol/L)*
D	*diffusion coefficient*
k_+^{ATP}, k_+^{CaM}, k_+^{TnC}, k_+^{Fluo4}	*Ca^{2+} on-rate constants for ATP, CaM, TnC and Fluo-4*
k_-^{ATP}, k_-^{CaM}, k_-^{TnC}, k_-^{Fluo4}	*Ca^{2+} off-rate constants for ATP, CaM, TnC and Fluo-4*
[*ATP*], [CaM], [*TnC*], [*Fluo4*]	*ATP, CaM, TnC and Fluo-4 concentrations (nmol/L)*
[*Ca.ATP*],[Ca.CaM],[*Ca.TnC*], [*Ca.Fluo4*]	*Ca^{2+}-ATP, Ca^{2+}-CaM, Ca^{2+}-TnC, Ca^{2+}-Fluo4 concentrations (nmol/L)*

A one-dimensional diffusion model equation (A) was initially built on a combination of Ca2+ release, Ca2+ uptake, Ca2+ diffusion, and Ca2+ interaction with principal cellular ligands (15). The model assumes an array of elements (nodes) that release Ca2+, and the propagation of free Ca2+ from node to node is dictated by diffusion (B) and interaction with cellular ligands (C). CICR activates adjacent Ca2+ release nodes. we assumed a pulse-shaped Ca2+ release flux (*R*) with exponential rise and fall (D). Opening of the Ca2+ channel was spontaneous or triggered by CICR when nodal [Ca2+] exceeded a variable threshold (*Thr*). Ca2+ release through open channels was assumed to exhibit a decay inversely proportional to [Ca2+]i near the channel. For simplification, we only considered the binding of free-Ca2+ with ATP, Fluo-4, calmodulin (CaM), and troponin C (TnC) (C). The ligand concentrations (*Ligand*) and rate constants (*k*) were adjusted to experimental temperature, assuming a Q10 of 2. We assumed that [Mg2+]i was constant (1 mM) and that ATP only binds Ca2+ and Mg2+. Ca2+ uptake nodes surrounded each Ca2+ release node and were located mid-distance between release nodes to mimic the location of Ca2+ pumps in the longitudinal SR. Ca2+ uptake flux (*U*) was assumed to follow Hill kinetics (E). Parameters of Ca2+ uptake by the SR were measured from cardiac myocytes in the laboratory. Cytosolic [Ca2+]i at rest was assumed to be 70 nM and the calculation begins with the buffers in equilibrium, using a physical diffusion coefficient D for Ca2+ (in water) of 3.0·10-6 cm2·s-1. The integration interval was 10-7 s. Meaning of model parmeters is indicated Panel F.

A more recent system of differential equations was developed from the same principles and was used for the numerical data of Figure 4. The calculations are detailed in [24,38] and *Matlab codes are available at:*
https://www.mun.ca/medicine/media/production/medicine/documents/Model_code.pdf
